# iLIR@viral: A web resource for LIR motif-containing proteins in viruses

**DOI:** 10.1080/15548627.2017.1356978

**Published:** 2017-08-14

**Authors:** Anne-Claire Jacomin, Siva Samavedam, Hannah Charles, Ioannis P. Nezis

**Affiliations:** School of Life Sciences, University of Warwick, Coventry, UK

**Keywords:** AIM, Atg8, database, LC3-interacting region motif, LIR, LIR-containing protein, LIRCP, LRS, prediction, virus

## Abstract

Macroautophagy/autophagy has been shown to mediate the selective lysosomal degradation of pathogenic bacteria and viruses (xenophagy), and to contribute to the activation of innate and adaptative immune responses. Autophagy can serve as an antiviral defense mechanism but also as a proviral process during infection. Atg8-family proteins play a central role in the autophagy process due to their ability to interact with components of the autophagy machinery as well as selective autophagy receptors and adaptor proteins. Such interactions are usually mediated through LC3-interacting region (LIR) motifs. So far, only one viral protein has been experimentally shown to have a functional LIR motif, leaving open a vast field for investigation. Here, we have developed the iLIR@viral database (http://ilir.uk/virus/) as a freely accessible web resource listing all the putative canonical LIR motifs identified in viral proteins. Additionally, we used a curated text-mining analysis of the literature to identify novel putative LIR motif-containing proteins (LIRCPs) in viruses. We anticipate that iLIR@viral will assist with elucidating the full complement of LIRCPs in viruses.

## Introduction

Autophagy is a multistep process that consists of the isolation of cytoplasmic components into double-membrane vesicles, called autophagosomes, that shuttle to lysosomes, which serve as end-point degradative organelles. It is a catabolic mechanism that enables the removal of damaged or excess cellular organelles and proteins, thereby contributing to the maintenance of cell homeostasis and survival.^1^

The autophagic machinery is highly conserved from unicellular eukaryotes to metazoans. Among the proteins that take part in this process, the Atg8-family proteins play a central role.^2^ Indeed, these proteins are involved in the elongation and maturation of the autophagosome and its fusion with lysosomes.^1^^,^^3^ Phosphatidylethanolamine-conjugated Atg8-family proteins reside on autophagosomal membranes where they can contribute to the recruitment of other core autophagy machinery proteins essential for the effective course of the autophagy process.^1^^,^^4-7^

Although originally considered to be a nonselective bulk degradation mechanism, a gathering body of evidence over the past decade suggests that autophagy is much more selective than initially appreciated. Selective targeting of cellular components to autophagosomes for degradation relies on the existence of selective autophagy receptors able to recognize and tether cargos toward nascent autophagosomes.^8^^,^^9^ Examples of selective autophagy include aggrephagy, mitophagy, lipophagy, and xenophagy.^10-13^ The interaction between selective autophagy receptors and Atg8-family proteins is essential for the proper steering of the cargo for degradation. These receptors typically contain an LC3-interacting region (LIR, also known as LRS, AIM or GIM; the latter correspond to LC3 recognition sequence, Atg8-interacting motif and GABARAP interaction motif respectively) critical for the binding to Atg8-family proteins.^7^^,^^14-25^ The LIR motif consists of a short amino acid sequence with a core motif originally described as W/F/YxxI/L/V (where ‘x’ represents any amino acids, and referred to as WxxL hereafter).^7^^,^^18^ This sequence has lately been relaxed and extended to 6 amino acids to integrate most of the experimentally verified LIRs. The new consensus sequence (called xLIR hereafter) is [ADEFGLPRSK][DEGMSTV]**[WFY]**[DEILQTV][ADEFHIKLMPSTV]**[ILV]**, where the residues in positions 3 and 6 correspond to the most crucial ones for the interaction with Atg8-family proteins.^26-28^

Besides its role in cellular homeostasis, autophagy is also involved in the innate immune response against pathogens.^13^^,^^29^^,^^30^ Recent years have seen an outburst of studies on autophagy and viral infections. Autophagy may exert a variety of antiviral functions, including the degradation of viral components (known as virophagy), the activation of innate immunity by the delivery of viral nucleic acids to the Toll-like receptors, and adaptive immunity through the major histocompatibility complex II (MCH-II/HLA class II), or the control of the production of reactive oxygen species (ROS).^31-43^ However, to be successful, viruses have evolved mechanisms to evade host defense. Several viruses have thus developed strategies to use the autophagy machinery or even thrive in the autophagosomes and promote their replication, spread, and survival.^44-47^ Other viruses susceptible to autophagy have evolved mechanisms to counteract autophagy activation by expressing proteins that interfere with the cellular machinery, essentially inhibiting the autophagosome-lysosome fusion or interfering with early stages of autophagy activation.^48-56^ A few proteins from viruses infecting mammals and plants that interfere with the host autophagy process have been shown to associate with Atg8-family proteins.^52^^,^^57-62^ Yet, only one LIR-dependent interaction has been reported.^62^

We have developed a database, iLIR@viral (http://ilir.uk/virus/), that organizes information on the presence of LIR motifs in viral proteins. Additionally, a curated text-mining analysis of the literature permitted us to predict functional LIR motifs in viral proteins that have already been shown to associate with Atg8-family members.

## Results and discussion

### Content of the iLIR@viral database

**Figure 1.**
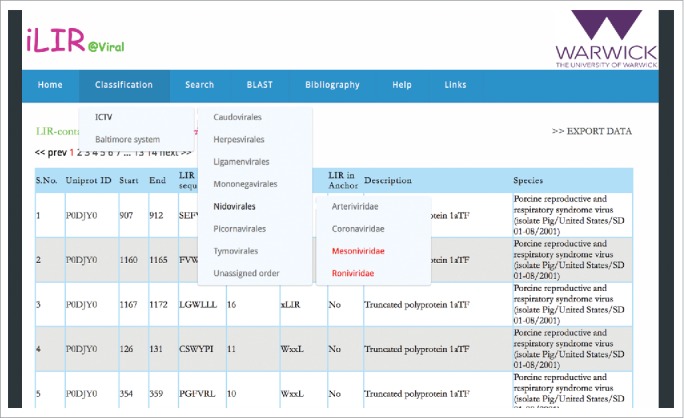
Screenshot of the iLIR@Viral database ‘Classification’ menu. Example for the ICTV classification system. The genera for which no LIRCPs were found appear in red.

The iLIR@viral database is a web resource freely available to the academic community at http://ilir.uk/virus/. Various functionalities are accessible under specific menus. The ‘Classification’ menu gives access to the complete list of putative LIR motifs in viral proteins. Two virus taxonomic systems have been used: the nomenclature used by the International Committee on Taxonomy of Viruses (ICTV) to name the species, genus and families of each of the viruses cited in the database, and the Baltimore classification system that groups viruses depending on their genome and kind of replication (dsDNA, dsRNA, ssDNA, ssRNA and reverse-transcribing viruses) (see Methods).^63^^,^^64^ For each specific family or group of viruses, the data are presented in a table containing (i) the clickable UniProtKB accession number of the protein, (ii) the information related to the LIR-motif (position, sequence, PSSM score, if the pattern is recognized as an xLIR or WxxL motif and the presence of the motif in an intrinsically disordered region (ANCHOR)), (iii) the name of the protein and (iv) the name of the species. For some genera, no putative LIR-containing protein (LIRCP) could be identified; for accuracy in the classification, these are listed but appear in red (Fig. 1).

The ‘Search’ menu allows the user to look in the database for a specific protein or virus order, family, subfamily, genus, species or common name. Uniprot identifiers can also be used in the search function.

The BLAST (Basic Local Alignment Search Tool) menu offers the user to search the database using a protein sequence as query against the reviewed set of viral proteins from UniProt database. We have used Position-Specific Iterative (PSI) BLAST to search against the database.^65^ This search can be run against any of the viral classification systems described above. By default, the BLAST search runs against all the data available in the database with a default e-value 0.01; nevertheless, the user has the possibility to run the BLAST search against a specific category (Baltimore classification) or Order taxonomic rank (ICTV classification), and define a different e-value. The results page displays the subject sequences from the database that match the query sequence. The LIR patterns are highlighted with red asterisks. The menus ‘Bibliography’ and ‘Help’ provide users with additional information. Finally, the ‘Links’ menu gives access to other iLIR web resources, inducing the iLIR search tool and the iLIR Database for eukaryote model organisms.^27^^,^^28^

### Analysis of the content of the database

**Figure 2.**
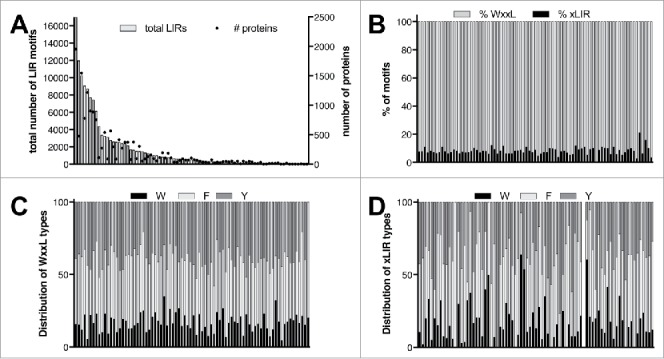
Distribution of LIR motifs in viruses. (A) Representation of the number of LIR motifs per family (bar chart, plotted on the left axis) and total number of reviewed sequences from UniProt (dot chart, plotted on the right axis). (B) Proportion of xLIR (black) and WxxL (gray) across the different families. (C) Distribution of the W-, F- and Y-type of WxxL patterns across the different families (see also Table S4). (D) Distribution of the W-, F- and Y-type of xLIR patterns across the different families (see also Table S4).

We have used the iLIR web resource (https://ilir.warwick.ac.uk) to identify LIRCPs in viruses (see Methods for details).^27^^,^^28^ Out of 16,609 reviewed viral sequences available from UniProt across 2569 individual viral species we found that 15,589 of them contain either xLIR or WxxL motifs. 6376 proteins contain xLIR motifs whereas 15,460 contain WxxL motifs. 6247 proteins contain both xLIR and WxxL motifs whereas 129 proteins contain only xLIR motifs (without containing WxxL patterns) and 9213 proteins contain only WxxL motifs (without xLIR patterns) (Table S1). We found a correlation between the total number of putative LIR motifs identified in a family and number of sequences (Fig. 2A). On average, we found 8.3 LIR motifs per sequence. The fact that viral sequences often refer to polyproteins instead of individual proteins is possibly an explanation of the high proportion of patterns identified.

The iLIR web resource can make the distinction between xLIR and WxxL patterns.^27^^,^^28^ We noticed that a vast majority of the motifs identified in viral proteins correspond to the WxxL pattern (Fig. 2B and Table S1). The identification of putative LIR motifs has been done for all the reviewed sequences for viral proteins. Among the sequences sorted as putative LIR-containing proteins, 1517 sequences belong to 188 species of bacteriophages (Table S2). However, it is likely that these sequences correspond to false-positive hits as bacteria don't have an autophagy process. Using a hypergeometric test (see supplementary information), we compared the enrichment fold of proteins containing LIR motifs (LIRCPs) in bacteriophages with viruses infecting eukaryotes. We noticed that the enrichment fold for both xLIR and WxxL patterns in LIRCPs was higher in viruses infecting eukaryotes than in bacteriophages (Table S2) which is in line with the fact that autophagy has been reported only in eukaryotes.

We have previously identified all the verified and putative LIR motifs in eukaryotic model organisms.^28^ We also compared the enrichment of putative LIR motifs in viruses infecting some of the model organisms (human, mouse, rat, and chicken) (Table S3). Two hypergeometric tests have been conducted to compare the enrichment fold of LIRCPs in viruses against the host: one for proteins containing all combination of LIR patterns (i.e., either xLIR or WxxL, or both kind) and another considering only the proteins containing at least one xLIR motif (i.e., xLIR patterns alone or along with one or more WxxL patterns). We observed that when all the possible combinations of LIR patterns are taken into account, there is an enrichment of putative LIR motifs in viruses compared with the host organism for all 4 model organisms tested. However, there is an enrichment of xLIR patterns in the host compared with the infecting viruses.

The LIR motifs can be divided into 3 subtypes depending on the residue at the first position: W-, F- and Y-types.^8^ It has been shown that the F-type LIR motif of mammalian ULK1 and ATG13 has a preference for GABARAP proteins, thus suggesting that the subtype of the LIR motifs could be related to a specificity toward Atg8-family proteins.^7^ Additionally F-type and Y-type LIR motifs are mostly contained in selective autophagy adaptor proteins.^8^ We thus analyzed the distribution of W-, F- and Y-type of WxxL and xLIR patterns at the viral order and family levels (Table S4 and Fig. 2C, D). We observed that 45% and 38% of the putative LIR motifs matching the WxxL pattern are of F-type and Y-type respectively. W-type motifs are the least represented, with about 17% of the patterns (Fig. 2C). Similar distribution could be observed for the putative xLIR motifs with a higher variability across families, probably due to the low representation of xLIR motifs compared with WxxL patterns (Fig. 2D).

### Manual literature curation for the identification of novel LIRCPs in viruses

To assess the trustworthiness of our in silico screening for LIRCPs in viruses, we compared our data with the already published viral proteins listed on the web resource ViralZone as modulators of autophagy.^66^ ViralZone classifies 180 entries related to the activation of the host autophagy, and 163 entries linked to the inhibition of host autophagy. We found all these entries in our database.

### Viruses inhibiting autophagy

Among viruses that inhibit autophagy, only 2 proteins have been shown to interact directly with mammalian Atg8-family proteins: Viral infectivity factor (Vif) from HIV-1 binds to all Atg8-family proteins, and matrix protein 2 (M2) from influenza was shown to bind to LC3. Yet, influenza M2 protein is the only one that contains a LIR motif that has been experimentally validated.^52^^,^^62^ Other viral proteins listed in ViralZone as being related to inhibition of the autophagy process are the neurovirulence factor ICP34.5 and RNA-binding protein US11 from human herpesviruses, the protein Nef from HIV-1, and the protein TRS1 from human cytomegalovirus.^49^^,^^61^^,^^62^^,^^67^ Negative regulation of autophagy by ICP34.5 and TRS1 proteins depends on their ability to interact with BECN1/Beclin 1;^68-70^ while US11 function has been linked to the protein kinase EIF2AK2/PKR.^71^ Putative, or functional, LIR motifs could be identified for all these proteins using the iLIR web resource, except for the RNA-binding protein US11.

The protein M2 from influenza A virus is necessary and sufficient for the inhibition of the autophagic degradation of the virus by blocking the fusion between the autophagosomes and lysosomes.^49^ These results were further confirmed and extended by Beale and colleagues who show that the cytoplasmic tail of M2 interacts in a LIR-dependent manner with LC3 and promotes the relocalization of LC3 at the plasma membrane.^62^ We have identified a WxxL motif at positions 89 to 94 that has the highest PSSM score (8 to 9), and corresponds to the one experimentally verified (FVSI).^27^^,^^72^ It is an F-type LIR motif and the fact that M2 protein has been shown to block the autophagosome-lysosome fusion suggests that it may act as a an adaptor protein.

We were also able to identify one xLIR motif (11-EGWQTI-16) in the sequence of the accessory viral protein Nef of the virus HIV-1 group M subtype B (strain 89.6). Nef colocalizes with LC3 and BECN1, and contributes to the inhibition of autophagic maturation, thus protecting the virus from elimination by autophagy.^61^^,^^67^

The proteins TRS1 from human cytomegalovirus and neurovirulence factor ICP34.5 from human herpesvirus (HSV) both interact with BECN1 via a specific BECN1-binding domain. This interaction is required for the inhibition of autophagosome maturation and fusion with the lysosomes.^68-70^^,^^73^ While iLIR could detect several WxxL motifs in TRS1 sequence, a single one in an intrinsically disordered region was identified for ICP34.5 whose sequence (64-RQWLHV-69) is quite well conserved among 4 strains of HSV-1 and one strain of HSV-2. However, to date, there is no evidence of association between ICP34.5 and LC3 proteins.

### Viruses activating autophagy

We observed that 7% of the reviewed sequences that contains at least one putative LIR motif correspond to the genome polyprotein from various viruses. Because polyproteins are processed co- and post-translationally by both host and viral proteases, we ran a systematic PubMed search with the terms ‘name of the virus + autophagy’ followed by ‘name of the virus + LC3’ to restrain the result outcome as necessary, finally we looked for papers (excluding reviews and commentaries) that specifically mention proteins derived from the processing of the viral genome polyprotein.

Our literature searching strategy pinpointed several nonstructural viral proteins; one of those was the protein NS1 from Dengue viruses. Studies have shown that NS1 protein from Dengue virus type 2 (DENV-2) colocalizes with LC3 and that DENV-2 particles and autophagosomes travel together during viral infection.^58^^,^^74^ In contrast to DENV-2, NS1 protein from DENV-3 displays a low level of colocalization with LC3.^75^ Sequence alignment of NS1 proteins from DENV-2 and DENV-3 showed that they are highly conserved. However, checking for the presence of LIR motifs revealed a discrepancy between them. We observed that DENV-2 NS1 has an xLIR motif (ASFIEV) that is not recognized in any DENV-3 NS1 sequences due to the substitution F to L, as well as an additional WxxL motif with a PSSM score 12 (RAWNSL) that is absent in DENV-3 (SL to VW) (Fig. S1). It is possible that the absence of either the xLIR or WxxL motifs (or both) in DENV-3 is responsible for its lower affinity to LC3.

Other nonstructural proteins from different viruses interact with LC3 proteins. For instance, the nonstructural protein NS5A from Hepatitis C virus that colocalizes and can be coimmunoprecipitated with LC3 proteins when ectopically expressed in various hepatoma cell lines.^59^^,^^76^^,^^77^ Also, the viral peptide 2BC and the protein 3A encoded by the genome polyprotein from Poliovirus type 1 interact with LC3-II.^60^^,^^78^ All these proteins contain WxxL motifs.

Finally, we were able to identify proteins from Zika virus that have been just recently related to autophagy. Independent studies have shown that Zika virus activates autophagy and that the formation of autophagosomes is crucial to the replication of the virus.^79-81^ It appears that the nonstructural proteins NS4A and NS4B are responsible for the induction of autophagy in infected cells by inhibiting the AKT-MTOR signaling, and both of them contain 3 WxxL motifs.^80^^,^^82^ Very little is known about the relation between Zika virus infection and autophagy modulation. We have found several proteins encoded by Zika genome polyprotein that contain LIR motifs, that could be good candidates for experimental validation.

## Conclusion

Autophagy is an evolutionarily conserved and highly regulated, intracellular catabolic mechanism that is essential for maintaining homeostasis and coping with nutrient starvation. It is increasingly appreciated that autophagy can be highly selective, and that xenophagy, the selective autophagy of pathogens, is an important aspect of the immune response, which protects against infection. A vast array of viruses are associated with autophagy, and we have found several viral proteins containing putative LIR motifs that are thought to interact with the autophagic machinery via Atg8-family proteins. A continued research effort to better understand how these viral proteins interact with the autophagic machinery may provide therapeutic strategies and ultimately lead to the discovery of novel pharmacological agents to fight viral infections.

## Methods

### Identification of putative LIR motifs in viral proteins

Protein sequences of all reviewed viral proteins were downloaded from Uniprot database vailable at: http://www.uniprot.org/ [Accessed 20 September 2016]. xLIR and WxxL patterns for these proteins were identified using the approach suggested previously.^27^^,^^28^ For a given protein, information related to the start and end of LIR pattern, actual LIR sequence, PSSM score (Position specific scoring matrix), similar LIRCPs and presence or absence xLIR and WxxL in intrinsically disordered region were obtained.

### Virus classification system

The taxonomic lineage obtained from UniProt for all the reviewed viral proteins correspond to the Baltimore classification system. To do the conversion between the Baltimore and ICTV classification systems, we matched the organism name for each reviewed sequence from UniProt with the organism names obtained from the ICTV master species list 2015 v1, available at: https://talk.ictvonline.org/files/master-species-lists/m/msl/5945 [Accessed 20 September 2016].

The differences between the classification systems were identified through a battery of SQL queries. The details of these are attached in Tables S5, S6 and S7. Levenshtein distance (also known as edit distance) was used to compare the species name that belongs to a particular genus and family between both the classification systems. Levenshtein distance is defined as the minimal number of characters required to replace, insert or delete to transform one string into another. The Levenshtein distance is symmetric and it holds:

Here, ‘x’ and ‘y’ are 2 strings and d(x,y) is the distance between ‘x’ and ‘y’ put as minimal cost of operations to transform ‘x’ to ‘y’. The complexity of the algorithm is O(m*n), where n and m are the lengths of 2 strings.^83^ We used Perl extension for approximate matching (Search.cpan. org, (2016). String-Approx-3.27. Retrieved from: http://search.cpan.org/CPAN/authors/id/J/JH/JHI/String-Approx-3.27.tar.gz) The closer the value of Levenshstein distance to zero, the closer are the species names.

Using this approach, we could reliably justify the ICTV classification of 14055 out 15589 proteins loaded into the database.

### Set up of Web-database application

To share the information beyond our MySQL (v5.6.33) DBMS (Database Management System), we built a website using HTML, CSS, JavaScript and PHP (v5.6.27) technologies. It is hosted at http://ilir.uk/virus. Through the website, users can navigate for LIRCPs using both Baltimore and ICTV Virus classification systems.

## Supplementary Material

Supplemental Files

## Abbreviations

AIM: Atg8-interacting motif
Atg8: autophagy-related 8
BLAST: Basic Local Alignment Search Tool
DENV: Dengue virus
HIV: human immunodeficiency virus
HSV: herpes simplex virus
ICP34.5: infected cell protein 34.5
ICTV: International Committee on Taxonomy of Viruses
LIR: LC3-interacting region
LIRCP: LIR-containing protein
LRS: LC3 recognition sequence
M2: matrix protein 2
MAP1LC3/LC3: microtubule-associated protein 1 light chain 3
Nef: Negative Regulatory Factor
NS: nonstructural protein
PSSM: position-specific scoring matrix
US11: unique short US11 glycoprotein
xLIR: extended LIR motif
