# Supplementary material for: iLIR@viral: A web resource for LIR motif-containing proteins in viruses
Source: Autophagy. 2017 Aug 14;13(10):1782–9. doi: 10.1080/15548627.2017.1356978 (PMC5640201; doi:10.1080/15548627.2017.1356978)
Supplement: Supplemental Files [file kaup-13-10-1356978-s001.zip › 2017AUTO0081R1-s02.docx]

**Supplementary information**

**iLIR@viral: a web resource for LIR motif-containing proteins in viruses**

Anne-Claire Jacomin^1,¶^, Siva Samavedam^1,¶^, Hannah Charles^1^ and Ioannis P. Nezis^1,*^

**Enrichment of LIR motifs in viruses infecting eukaryotes compared to bacteriophages**

A hypergeometric test has been done with the following parameters for xLIR patterns:

1) All proteins with xLIR/WxxL motif from phages (success of sample)

2) Total proteins of phages (sample population)

3) All proteins with xLIR/WxxL motif from all viruses (success in population)

4) Total proteins of all viruses (population)

The results indicate that for proteins with xLIR motifs, both bacteriophages and viruses infecting eukaryotes were underenriched compared to expectations. However, the enrichment fold was lesser in viruses infecting eukaryotes (1.05 fold vs 1.25 fold for phages) and hence it was slightly better than prokaryotes.

In the case of WxxL motif-containing proteins, while phages were underenriched compared to expectations by a fold of 1.02, viruses infecting eukaryotes were enriched by a fold of 1.

The details of these tests along with *P* values are attached supportingdata.xlsx file. Please see the sheet ‘Phages_vs_eukaryotes’ for more details. (We used green color to indicate underenrichment fold value and red color to indicate overenrichment fold value)

**Figure S1.** CLUSTAL O(1.2.2) multiple sequence alignment of NS1 from Dengue viruses DENV-1 to -4. xLIR/WxxL motifs are highlighted, the colour is representative of the DENV stereotype: DENV-1, DENV-2, DENV-3, DENV-4. Changes in amino acid resulting in either no effect (i.e., LIR motif still detected by iLIR) or loss of the detection of the LIR motif are shown in **bold**.

sp|Q58HT7|775-1126 DTGCAVSWSGKELKCGSGIFVIDNVHTWTEQYKFQPESPARLASAILNAHEDGVCGIRST

sp|Q2YHF0|775-1126 DMGCAVSWSGKELKCGSGIFVIDNVHTWTEQYKFQPESPARLASAILNAHKDGVCGIRST

sp|Q2YHF2|775-1126 DMGCVVSWTGKELKCGSGIFVTDNVHTWTEQYQFQPESPARLASAILNAHKDGVCGIRST

sp|P09866|775-1126 DMGCVASWSGKELKCGSGIFVVDNVHTWTEQYKFQPESPARLASAILNAHKDGVCGIRST

sp|Q5UCB8|775-1126 DMGCVVSWSGKELKCGSGIFVADNVHTWTEQYKFQPESPARLASAILNAHKDGVCGIRST

sp|Q9WDA6|776-1127 DSGCVVSWKNKELKCGSGIFVTDNVHTWTEQYKFQPESPSKLASAIQKAHEEGICGIRSV

sp|P12823|776-1127 DSGCVVSWKNKELKCGSGIFVTDNVHTWTEQYKFQPESPSKLASAIQKAHEEGICGIRSV

sp|P07564|776-1127 DSGCVVSWKNKELKCGSGIFITDNVHTWTEQYKFQPESPSKLASAIQKAHEEGICGIRSV

sp|P14340|776-1127 DSGCVVSWKNKELKCGSGIFITDNVHTWTEQYKFQPESPSKLASAIQKAHEEGICGIRSV

sp|P14337|776-1127 DSGCVVSWKNKELKCGSGIFITDNVHTWTEQYKFQPESPSKLASAIQKAQEEGICGIRSV

sp|P29991|776-1127 DSGCVVSWKNKELKCGSGIFITDNVHTWTEQYKFQPESPSKLASAIQKAHEEDICGIRSV

sp|P29990|776-1127 DSGCVVSWKNKELKCGSGIFITDNVHTWTEQYKFQPESPSKLASAIQKAHEEGICGIRSV

sp|Q5UB51|774-1125 DMGCVINWKGKELKCGSGIFVTNEVHTWTEQYKFQADSPKRLATAIAGAWENGVCGIRST

sp|Q6YMS4|774-1125 DMGCVINWKGKELKCGSGIFVTNEVHTWTEQYKFQADSPKRLATAIAGAWENGVCGIRST

sp|Q6YMS3|774-1125 DMGCVINWKGKELKCGSGIFVTNEVHTWTEQYKFQADSPKRLATAIAGAWENGVCGIRST

sp|Q99D35|774-1125 DMGCVINWKGKELKCGSGIFVTNEVHTWTEQYKFQADSPKRLATAIAGAWENGVCGIRST

sp|P27915|774-1125 DMGCVINWKGKELKCGSGIFVTNEVHTWTEQYKFQADSPKRVATAIAGAWENGVCGIRST

sp|P27909|776-1127 DSGCVINWKGRELKCGSGIFVTNEVHTWTEQYKFQADSPKRLSAAIGRAWEEGVCGIRSA

sp|P17763|776-1127 DSGCVINWKGRELKCGSGIFVTNEVHTWTEQYKFQADSPKRLSAAIGKAWEEGVCGIRSA

sp|P33478|775-1126 DSGCVINWKGRELKCGSGIFVTNEVHTWTEQYKFQADSPKRLSAAIGKAWEEGVCGIRSA

* **. .*. :*********: ::********:** :** ::::** * :: :*****.

sp|Q58HT7|775-1126 TRLEN**I**MWKQITNELNYVLWEGGHDLTVVAGDVKGVLSKGKRALAPPVNDLKYSWKTWGK

sp|Q2YHF0|775-1126 TRLENVMWKQITNELNYVLWEGGHDLTVVAGDVKGVLSKGKRALAPPVNDLKYSWKTWGK

sp|Q2YHF2|775-1126 TRLENVMWKQITNELNYVLWEGGHDLTVVAGDVKGVLVKGKRALTPPVNDLKYSWKTWGK

sp|P09866|775-1126 TRLENVMWKQITNELNYVLWEGGHDLTVVAGDVKGVLTKGKRALTPPVSDLKYSWKTWGK

sp|Q5UCB8|775-1126 TRLENVMWKQITNELNYVLWEGGHDLTVVAGDVKGVLTKGKRALTPPVNDLKYSWKTWGK

sp|Q9WDA6|776-1127 TRLENLMWKQITSELNHILSENEVKLTIMTGDIKGIMQVGKRSLRPQPTELRYSWKTWGK

sp|P12823|776-1127 TRLENLMWKQITSELNHILSENEVKLTIMTGDIKGIMQVGKRSLRPQPTELRYSWKTWGK

sp|P07564|776-1127 TRLENLMWKQITPELNHILSENEVKLTIMTGDIKGIMQAGKRSLRPQPTELKYSWKTWGK

sp|P14340|776-1127 TRLENLMWKQITPELNHILSENEVKLTIMTGDIKGIMQAGKRSLQPQPTELKYSWKTWGK

sp|P14337|776-1127 TRLENLMWKQITPELNHILAENEVKLTIMTGDIKGIMQAGKRSLRPQPTELKYSWKTWGK

sp|P29991|776-1127 TRLENLMWKQITPELNHILSENEVKLTIMTGDIKGIMQAGKRSLRPQPTELKYSWKTWGK

sp|P29990|776-1127 TRLENLMWKQITPELNHILSENEVKLTIMTGDIKGIMQAGKRSLRPQPTELKYSWKTWGK

sp|Q5UB51|774-1125 TRMENLLWKQIANELNYILWENNIKLTVVVGDIIGVLEQGKRTLTPQPMELKYSWKTWGK

sp|Q6YMS4|774-1125 TRMENLLWKQIANELNYILWENNIKLTVVVGDTLGVLEQGKRTLTPQPMELKYSWKTWGK

sp|Q6YMS3|774-1125 TRMENLLWKQIANELNYILWENNIKLTVVVGDIIGVLEQGKRTLTPQPMELKYSWKTWGK

sp|Q99D35|774-1125 TRMENLLWKQIANELNYILWENNIKLTVVVGDITGVLEQGKRTLTPQPMELKYSWKTWGK

sp|P27915|774-1125 TRMENLLWKQIANELNYILWENDIKLTVVVGDITGVLEQGKRTLTPQPMELKYSWKTWGL

sp|P27909|776-1127 TRLENIMWKQISNELNHILLEND**I**KFTVVVGNANGILAQGKKMIRPQPMEHKYSWKSWGK

sp|P17763|776-1127 TRLENIMWKQISNELNHILLENDMKFTVVVGDVSGILAQGKKMIRPQPMEHKYSWKSWGK

sp|P33478|775-1126 TRLENIMWKQISNELNHILLENDMKFTVVVGDVVGILAQGKKMIRPQPMEHKYSWKSWGK

**:**::****: ***::* * .:*::.*: *:: **: : * : :****:**

sp|Q58HT7|775-1126 AKIFTPEAKNSTFLIDGPDTSECPNERRAWNFLEVEDYGFGMFTTNIWMKFREGSSEVCD

sp|Q2YHF0|775-1126 AKIFTPETRNSTFLVDGPDTSECPNERRAWNFLEVEDYGFGMFTTNIWMKFREGSSEVCD

sp|Q2YHF2|775-1126 AKIFTPEAKNSTFLIDGPDTSECPNERRAWNFLEVEDYGFGMFTTSIWMKFREGSSEVCD

sp|P09866|775-1126 AKIFTPEARNSTFLIDGPDTSECPNERRAWN**S**LEVEDYGFGMFTTNIWMKFREGSSEVCD

sp|Q5UCB8|775-1126 AKIFTPEARNSTFLIDGPDTSECPNERRAWNFLEVEDYGFGMFTTNIWMKFREGSSEVCD

sp|Q9WDA6|776-1127 AKMLSTELHNQTFLIDGPETAECPNTNRAWNSLEVEDYGFGVFTTNIWL**R**LREKQDAFCD

sp|P12823|776-1127 AKMLSTELHNQTFLIDGPETAECPNTNRAWNSLEVEDYGFGVFTTNIWL**R**LREKQDAFCD

sp|P07564|776-1127 AKMLSTESHNQTFLIDGPETAECPNTNRAWNSLEVEDYGFGVFTTNIWLKLREKQDVFCD

sp|P14340|776-1127 AKMLSTESHNQTFLIDGPETAECPNTNRAWNSLEVEDYGFGVFTTNIWLKLREKQDVFCD

sp|P14337|776-1127 AKMLSTESHNQTFLIDGPETAECPNTNRAWNSLEVEDYGFGVFTTNIWLKLKEKQDAFCD

sp|P29991|776-1127 AKMLSTESHNQTFFIDGPETAECPNTNRAWNSLEVEDYGFGVFTTNIWLKLKEKQDVFCD

sp|P29990|776-1127 AKMLSTESHNQTFLIDGPETAECPNTNRAWNSLEVEDYGFGVFTTNIWLKLKEKQDVFCD

sp|Q5UB51|774-1125 AKIVTAETQNSSFIIDGPNTPECPSASRAWN**VW**EVEDYGFGVFTTNIWLKLREVYTQ**S**CD

sp|Q6YMS4|774-1125 AKIVTAETQNSSFIIDGPNTPECPSASRAWN**VW**EVEDYGFGVFTTNIWLKLREVYTQLCD

sp|Q6YMS3|774-1125 AKIVTAETQNSSFIIDGPNTPECPSASRAWN**VW**EVEDYGFGVFTTNIWLKLREVYTQLCD

sp|Q99D35|774-1125 AKIVTAETQNSSFIIDGPSTPECPSASRAWN**VW**EVEDYGFGVFTTNIWLKLREVYTQLCD

sp|P27915|774-1125 AKIVTAETQNSSFIIDGPSTPECPSASRAWN**VW**EVEDYGFGVFTTNIWLKLREVYTQLCD

sp|P27909|776-1127 AKIIGADIQNTTFIIDGPDTPECPDEQRAWNIWEVEDYGFGIFTTNIWLKLRDSYTQMCD

sp|P17763|776-1127 AKIIGADVQNTTFIIDGPNTPECPDNQRAWNIWEVEDYGFGIFTTNIWLKLRDSYTQVCD

sp|P33478|775-1126 AKIIGADIQNTTFIIDGPDTPECPDDQRAWNIWEVEDYGFGIFTTNIWLKLRDSYTQMCD

**:. : :* :*::***.* ***. **** ********:***.**::::: **

sp|Q58HT7|775-1126 HRLMSAAIKDQKAVHADMGYWIESSKNQTWQIEKAS**L**IEVKTCLWPKTHTLWSNGVLESQ

sp|Q2YHF0|775-1126 HRLMSAAIKDQKAVHADMGYWIESSKNQTWQIEKAS**L**IEVKTCLWPKTHTLWSNGVLESQ

sp|Q2YHF2|775-1126 HRLMSAAIKDQKAVHADMGYWLESSKNQTWQIEKAS**L**IEVKTCLWPKTHTLWSNGVLESQ

sp|P09866|775-1126 HRLMSAAIKDQKAVHADMGYWIESSKNQTWQIEKAS**L**IEVKTCLWPKTHTLWSNGVLESQ

sp|Q5UCB8|775-1126 HRLMSAAIKDQKAVHADMGYWIESSKNQTWQIEKAS**L**IEVKTCLWPKTHTLWSNGVLESQ

sp|Q9WDA6|776-1127 SKLMSAAIKDNRAVHADMGYWIESALNDTWKIEKASFIEVKSCHWPKSHTLWSNGVLESE

sp|P12823|776-1127 SKLMSAAIKDNRAVHADMGYWIESALNDTWKIEKASFIEVKSCHWPKSHTLWSNGVLESE

sp|P07564|776-1127 SKLMSAAIKDNRAVHADMGYWIESALNDTWKMEKASFIEVKSCHWPKSHTLWSNGVLESE

sp|P14340|776-1127 SKLMSAAIKDNRAVHADMGYWIESALNDTWKIEKASFIEVKSCHWPKSHTLWSNGVLESE

sp|P14337|776-1127 SKLMSAAIKDNRAVHADMGYWIESALNDTWKIEKASFIEVKNCHWPKSHTLWSNGVLESE

sp|P29991|776-1127 SKLMSAAIKDNRAVHADMGYWIESALNDTWKIEKASFIEVKNCHWPKSHTLWSNGVLESE

sp|P29990|776-1127 SKLMSAAIKDNRAVHADMGYWIESALNDTWKIEKASFIEVKNCHWPKSHTLWSNGVLESE

sp|Q5UB51|774-1125 HRLMSAAIKDERAVHADMGYWIESQKNGSWKLEKASFIEVKTCTWPKSHTLWSNGVLESD

sp|Q6YMS4|774-1125 HRLMSAAVKDERAVHADMGYWIESQKNGSWKLEKAS**L**IEVKTCTWPKSHTLWTNGVLESD

sp|Q6YMS3|774-1125 HRLMSAAVKDERAVHADMGYWIESQKNGSWKLEKAS**L**IEVKTCTWPKSHTLWSNGVLESD

sp|Q99D35|774-1125 HRLMSAAVKDERAVHADMGYWIESQKNGSWKLEKAS**L**IEVKTCTWPKSHTLWSNGVLESD

sp|P27915|774-1125 HRLMSAAVKDERAVHADMGYWIESQKNGSWKLEKAS**L**IEVKTCTWPKSHTLWSNGVLESD

sp|P27909|776-1127 HRLMSAAIKDSKAVHADMGYWIESEKNETWKLARASFIEVKTCIWPKSHTLWSNGVLESE

sp|P17763|776-1127 HRLMSAAIKDSKAVHADMGYWIESEKNETWKLARASFIEVKTCIWPKSHTLWSNGVLESE

sp|P33478|775-1126 HRLMSAAIKDSKAVHADMGYWIESEKNETWKLARASFIEVKTCVWPKSHTLWSNGVLESE

:*****:**.:*********:** * :*:: :**:****.* ***:****:******:

sp|Q58HT7|775-1126 MLIPKAYAGPFSQHNYRQGYATQTVGPWHLGKLEIDFGECPGTTVTIQEDCDHRGPSLRT

sp|Q2YHF0|775-1126 MLIPKAYAGPISQHNYRQGYATQTVGPWHLGKLEIDFGECPGTTVTIQEDCDHRGPSLRT

sp|Q2YHF2|775-1126 MLIPKAYAGPFSQHNYRQGYATQTMGPWHLGKLEIDFGECPGTTVTIQEDCDHRGPSLRT

sp|P09866|775-1126 MLIPKSYAGPFSQHNYRQGYATQTVGPWHLGKLEIDFGECPGTTVTIQEDCDHRGPSLRT

sp|Q5UCB8|775-1126 MLIPKSYAGPFSQHNYRQGYATQTVGPWHLGKLEIDFGECPGTTVTIQEDCDHRGPSLRT

sp|Q9WDA6|776-1127 MVIPKNIAGPVSQHNNRPGYHTQTAGPWHLGKLEMDFDFCEGTTVVVTEECGNRGPSLRT

sp|P12823|776-1127 MVIPKNFAGPVSQHNNRPGYHTQTAGPWHLGKLEMDFDFCEGTTVVVTEDCGNRGPSLRT

sp|P07564|776-1127 MIIPKNFAGPVSQHNYRPGYHTQTAGPWHLGKLEMDFDFCEGTTVVVTEDCGNRGPSLRT

sp|P14340|776-1127 MIIPKNFAGPVSQHNYRPGYHTQTAGPWHLGKLEMDFDFCEGTTVVVTEDCGNRGPSLRT

sp|P14337|776-1127 MIIPKNLAGPVSQHNYRPGYHTQIAGPWHLGKLEMDFDFCDGTTVVVTEDCGNRGPSLRT

sp|P29991|776-1127 MIIPKNLAGPVSKHNYRPGYHTQITGPWHLGKLEMDFDFCDGTTVVVTEDCGNRGPSLRT

sp|P29990|776-1127 MIIPKNLAGPVSQHNYRPGYHTQITGPWHLGKLEMDFDFCDGTTVVVTEDCGNRGPSLRT

sp|Q5UB51|774-1125 MIIPKSLAGPISQHNHRPGYHTQTAGPWHLGKLELDFNYCEGTTVVITENCGTRGPSLRA

sp|Q6YMS4|774-1125 MIIPKSLAGPISQHNYRPGYHTQTAGPWHLGKLELDFNYCEGTTVVITESCGTRGPSLRT

sp|Q6YMS3|774-1125 MIIPKSLAGPISQHNHRPGYHTQTAGPWHLGKLELDFNYCEGTTVVITENCGTRGPSLRT

sp|Q99D35|774-1125 MIIPKSLAGPISQHNHRPGYHTQTAGPWHLGKLELDFNYCEGTTVVISENCGTRGPSLRT

sp|P27915|774-1125 MIIPKSLAGPISQHNHRPGYHTQTAGPWHLGKLELDFNYCEGTTVVISENCGTRGPSLRT

sp|P27909|776-1127 MIIPKMYGGPISQHNYRPGYFTQTAGPWHLGKLELDFDLCEGTTVVVDEHCGSRGPSLRT

sp|P17763|776-1127 MIIPKIYGGPISQHNYRPGYFTQTAGPWHLGKLELDFDLCEGTTVVVDEHCGNRGPSLRT

sp|P33478|775-1126 MIIPKIYGGPISQHNYRPGYFTQTAGPWHLGKLELDFDLCEGTTVVVDEHCGNRGPSLRT

*:*** .**.*:** * ** ** *********:** * ****.: * * ******:

sp|Q58HT7|775-1126 TTASGKLVTQWCCRSCTMPPLRFLGEDGCWYGMEIRPLSEKEENMVKSQVSA

sp|Q2YHF0|775-1126 TTASGKLVTQWCCRSCTMPPLRFLGEDGCWYGMEIRPLNEKEENMVKSQVSA

sp|Q2YHF2|775-1126 TTASGKLVTQWCCRSCTMPPLRFLGEDGCWYGMEIRPLSEREENMVKSQVSA

sp|P09866|775-1126 TTASGKLVTQWCCRSCTMPPLRFLGEDGCWYGMEIRPLSEKEENMVKSQVTA

sp|Q5UCB8|775-1126 TTASGKLVTQWCCRSCTMPPLRFLGEDGCWYGMEIRPLSEKEENMVKSQVTA

sp|Q9WDA6|776-1127 TTASGKLITEWCCRSCTLPPLRYRGEDGCWYGMEIRPLKEKEENLVSSLVTA

sp|P12823|776-1127 TTASGKLITEWCCRSCTLPPLRYRGEDGCWYGMEIRPLKEKEENLVSSLVTA

sp|P07564|776-1127 TTASGKLITEWCCRSCTLPPLRYRGEDGCWYGMEIRPLKEKEENLVNSLVTA

sp|P14340|776-1127 TTASGKLITEWCCRSCTLPPLRYRGEDGCWYGMEIRPLKEKEENLVNSLVTA

sp|P14337|776-1127 TTASGKLITEWCCRSCTLPPLRYRGEDGCWYGMEIRPLKEKEENLVNSLVTA

sp|P29991|776-1127 TTASGKLITEWCCRSCTLPPLRYRGEDGCWYGMEIRPLKEKEENLVNSLVTA

sp|P29990|776-1127 TTASGKLITEWCCRSCTLPPLRYRGEDGCWYGMEIRPLKEKEENLVNSLVTA

sp|Q5UB51|774-1125 TTVSGKLIHEWCCRSCTLPPLRYMGEDGCWYGMEIRPVNEKEENMVKSLVSA

sp|Q6YMS4|774-1125 TTVSGKLIHEWCCRSCTLPPLRYMGEDGCWYGMEIRPISEKEENMVKSLVSA

sp|Q6YMS3|774-1125 TTVSGKLIHEWCCRSCTLPPLRYMGEDGCWYGMEIRPISEKEENMVKSLVSA

sp|Q99D35|774-1125 TTVSGKLIHEWCCRSCTLPPLRYMGEDGCWYGMEIRPINEKEENMVKSLASA

sp|P27915|774-1125 TTVSGKLIHEWCCRSCTLPPLRYMGEDGCWYGMEIRPINEKEENMVKSLASA

sp|P27909|776-1127 TTVTGKIIHEWCCRSCTLPPLRFRGEDGCWYGMEIRPVKEKEENLVRSMVSA

sp|P17763|776-1127 TTVTGKTIHEWCCRSCTLPPLRFKGEDGCWYGMEIRPVKEKEENLVKSMVSA

sp|P33478|775-1126 TTVTGKIIHEWCCRSCTLPPLRFKGEDGCWYGMEIRPVKEKEENLVKSMVSA

**.:** : :*******:****: *************:.*:***:* * .:*ƒ

**Table S1.** Basic statistics on the database.

**Table S2.** Comparison of the distribution of the LIR patterns and enrichment fold in bacteriophages and viruses infecting eukaryotes.

A hypergeometric test has been used to determine the enrichment folds compared to expectation. Green and red colors indicate underenrichment and overenrichment folds, respectively.

**Table S3.** Comparison of the enrichment fold of putative LIR motifs in viruses and their host (human, mouse, rat and chicken).

A hypergeometric test has been used to determine the enrichment folds compared to expectation. Green and red colors indicate underenrichment and overenrichment folds, respectively.

**Table S4.** Number of putative LIR motifs (xLIR and WxxL patterns) for each family of viruses according to their type (W-, F- and Y-type).

**Table S5.** Comparison between ICTV and Baltimore classification systems.

‘Assigned’ means that a virus species has been assigned to a family or genus; ‘unassigned’ means that a virus species has not been assigned to a family or genera and thus Taxon name itself is ‘Unassigned’.

**Table S6.** List of Families and genera represented either on ICTV or Baltimore classification system only.

* indicates that these genera may be represented in ICTV with subclassified names.

**Table S7.** Details on the conversion of the classification between the Baltimore and ICTV systems.

Number highlighted in green and orange corresponds to the number of names assigned or unassigned at the order, family or genera levels. The top row for each classification system gives the total of orders, families, genera and species for bot assigned and unassigned names. For example, in the ICTV classification, there are 114 families of which 31 (29+2) belong to orders with assigned names (7) and 83 (82+1) belong to order (1) with unassigned name.
